# Roflumilast-N-oxide Induces Surfactant Protein Expression in Human Alveolar Epithelial Cells Type II

**DOI:** 10.1371/journal.pone.0038369

**Published:** 2012-07-16

**Authors:** Kerstin Höhne, Stephan J. Schließmann, Andreas Kirschbaum, Till Plönes, Joachim Müller-Quernheim, Hermann Tenor, Gernot Zissel

**Affiliations:** 1 Division of Internal Medicine, Department of Pneumology, University Medical Centre, Freiburg, Germany; 2 Faculty of Biology, University of Freiburg, Freiburg, Germany; 3 Division of Surgery, Department of Thoracic Surgery, University Medical Centre, Freiburg, Germany; 4 Nycomed GmbH Global Discovery, Nycomed GmbH, Konstanz, Germany; University of Tübingen, Germany

## Abstract

Surfactant proteins (SPs) are important lipoprotein complex components, expressed in alveolar epithelial cells type II (AEC-II), and playing an essential role in maintenance of alveolar integrity and host defence. Because expressions of SPs are regulated by cyclic adenosine monophosphate (cAMP), we hypothesized that phosphodiesterase (PDE) inhibitors, influence SP expression and release. Analysis of PDE activity of our AEC-II preparations revealed that PDE4 is the major cAMP hydrolysing PDE in human adult AEC-II. Thus, freshly isolated human AEC-II were stimulated with two different concentrations of the PDE4 inhibitor roflumilast-N-oxide (3 nM and 1 µM) to investigate the effect on SP expression. SP mRNA levels disclosed a large inter-individual variation. Therefore, the experiments were grouped by the basal SP expression in low and high expressing donors. AEC-II stimulated with Roflumilast-N-oxide showed a minor increase in SP-A1, SP-C and SP-D mRNA mainly in low expressing preparations. To overcome the effects of different basal levels of intracellular cAMP, cyclooxygenase was blocked by indomethacin and cAMP production was reconstituted by prostaglandin E2 (PGE2). Under these conditions SP-A1, SP-A2, SP-B and SP-D are increased by roflumilast-N-oxide in low expressing preparations. Roflumilast-N-oxide fosters the expression of SPs in human AEC-II via increase of intracellular cAMP levels potentially contributing to improved alveolar host defence and enhanced resolution of inflammation.

## Introduction

Pulmonary surfactant is composed of a variety of different lipids and four surfactant associated proteins (SP-A, SP-B, SP-C and SP-D) and is important for reduction of the surface tension at the air-liquid interface of the lung and participates in immunomodulatory processes. Alveolar epithelial cells type II are the main source expressing, synthesizing, and secreting all surfactant components [1], [2].

The most abundant surfactant protein in pulmonary surfactant is SP-A. Functional SP-A is important for secretion, synthesis and recycling of surfactant phospholipids and involved in innate immune reactions. SP-D regulates lipid homeostasis and some effects of SP-A. Both, SP-A and SP-D, bind to a variety of pathogens, acting as an opsonin to enhance phagocytosis and support pulmonary clearance. Besides these immune defence tasks, they disclose anti-inflammatory effects and play a role in the resolution of pulmonary inflammation [3].

SP-B and SP-C are essential for reduction of surface tension and stabilization of mammalian surfactant lipids [4]. SP-B is required for normal lung function and SP-C imparts important surface properties of surfactant phospholipid mixtures. Mutations in SP-C are associated with chronic parenchymal lung disease [5]. Both are components of surfactant mixture used for the treatment of the respiratory distress syndrome (RDS) in infants.

SP-B, SP-C and SP-D are encoded by single genes [5], [6], [7] with different isoforms. Human SP-A is encoded by two functional genes, *SFTPA1* coding for SP-A1 and *SFTPA2* coding for SP-A2 and one SP-A pseudogen [8].

It has been shown that in foetal lungs SP-A, SP-B, SP-C and SP-D mRNA are regulated by intracellular adenosine 3̀, 5̀-cyclic monophosphate (cAMP) [9], [10]. Cyclic AMP is generated by adenylyl cyclase secondary to activation of heterotrimeric Gs protein by ligated G-Protein coupled receptors (GPCRs) such as the β-adrenergic or prostaglandin E2 receptor (EP2) yet degraded by the superfamily of cyclic nucleotide phosphodiesterases (PDE). Among the eleven PDE families described in mammals PDE4 has gained attraction. PDE4 specifically hydrolyzes cAMP and is expressed in inflammatory and structural cells. PDE4 inhibitors exert anti-inflammatory effects and the selective PDE4 inhibitor Roflumilast has been suggested for treatment of COPD or asthma [11], [12], [13]. Roflumilast was approved for maintenance treatment of severe COPD in the European Union [14]. During metabolism roflumilast is converted into its active form, roflumilast-N-oxide, sharing high potency and selectivity with the parent compound [14]. Whether human adult AEC-II are targeted by PDE4 inhibitors remained unexplored.

The current study was designed to unravel potential effects of the PDE4 inhibitor roflumilast-N-oxide on SP-A1, SP-A2, SP-B, SP-C and SP-D in isolated adult human primary AEC-II.

## Methods

### Patients

The study was approved by the Ethics Committee of the University Medical Centre Freiburg. The material used for isolation of the cells was selected by AK and TP after lobectomy or pneumectomy due to lung cancer. Written informed consent from all participants involved was obtained.

In total, macroscopically normal lung tissue samples were obtained from pulmonary resections of 38 patients with primary lung cancer undergoing partial resection, lobectomy or pneumonectomy. The fact that in histological examination all tumours were surrounded by normal tissue suggests that the used samples were free of malignant cells. 25 patients were current smokers; seven patients were ex-smokers and one patient never smoked (5 unknown). Mean smoking duration of the smokers was 39±22 pack-years (14 unknown). Mean age of the patients was 62±10 years. 29 patients were male and nine were female.

Based on median relative surfactant protein mRNA expression the donors were defined as “low” or “high” surfactant expressing donors for statistical analysis (below median  =  low; above median  =  high).

### Isolation of Human Primary AEC-II

AEC-II were isolated as described previously [15], with some modifications [16]. In brief, macroscopically tumour-free lung tissue was first sliced, washed three times at 4°C in phosphate-buffered saline (PBS) and then digested in sterile dispase solution (2,5 mg dispase II (Invitrogen, Karlsruhe, Germany) per mL and 50 µg/mL DNase I (Roche Diagnostics, Mannheim, Germany)) at 37°C for 60 minutes. After dispase digestion, the slices were thoroughly pipetted for several minutes using a 10 mL pipette with a wide inlet. Crude tissue and cell suspensions were filtered through nylon gauze with meshes of 50 and 20 µm. The cell suspension was then layered onto a density gradient solution (PAN Biotech GmbH, Aidenbach, Germany) and centrifuged at 800×g for 20 min. The cells of the interphase were washed and incubated in 100 mm Petri dishes (max. 6×10^7^ cells/dish) in RPMI 1640 (Invitrogen) with 10% foetal bovine serum (FBS Gold; PAA Laboratories, Cölbe, Germany) and 1% penicillin/streptomycin (Biochrom, Berlin, Germany) at 37°C in humidified incubator (5% CO2, 37°C) for 15, 20 and if necessary 30 minutes to remove adherent (mostly alveolar macrophages, monocytes and fibroblasts) from non-adherent cells.

Non-adherent cells were collected and counted. Cell viability was assessed using the trypan-blue exclusion method. After washing two times, the cells were further separated by anti-CD45-coated magnetic beads (Miltenyi Biotec, Bergisch Gladbach, Germany) using appropriate depletion columns as suggested by the supplier to remove remaining monocytes/macrophages and lymphocytes. Identity of the negatively fractioned AEC-II was confirmed by a modified Papanicolaou staining. The percentage of Papanicolaou-positive AEC-II was always >90%. The isolated AECII were resuspended and washed one time in culture medium. Preliminary experiments showed a decrease in surfactant protein expression over time, therefore we decided not to exceed 24 h of culture.

### Culture of AEC-II

AEC-II were cultured in RPMI 1640 with 10% FBS and 1% antibiotics (50 U/mL penicillin and 50 µg/mL streptomycin). To assess the effect of cAMP on surfactant protein expression AEC-II (2×10^6^ cells·mL^–1^) were treated with dibutyryl-cAMP (dbcAMP) and roflumilast-N-oxide (Nycomed, Konstanz, Germany) with or without 1 µM indomethacin and 10 nM prostaglandin E2 (PGE2) in 24 well plates at 37°C for 24 h. Attempts to measure surfactant proteins by ELISA or to perform Western blot directly from the cell culture supernatant failed. We therefore added 10 µg/ml Brefeldin (Sigma, St. Louis, MO, USA) to the cell cultures to trap the surfactant proteins within the cells. After supernatant removal, cells were lysed using 350 µl of “RLT plus lysis buffer” (Qiagen, Hilden, Germany) for RNA isolation resp. 200 µl lysis buffer (100 mM NaCl, 50 mM TrisHCl (pH7,6), 2 mM EDTA, 2 mM EGTA, 0.1% Triton-x-100, 1% protease inhibitor mix (BioVision, CA, USA) for Western blot.

### PDE Measurement

Human AEC-II cells (3×10^6^ cells) were washed twice in phosphate buffered saline (4°C) and resuspended in 1 mL homogenization buffer (137 mM NaCl, 2.7 mM KCl, 8.1 mM Na_2_HPO_4_, 1.5 mM KH_2_PO_4_, 10 mM Hepes, 1 mM EDTA, 1 mM MgCl_2_, 1 mM ß-mercaptoethanol, 5 µM pepstatin A, 10 µM leupeptin, 50 µM phenylmethylsulfonyl fluoride, 10 µM soybean trypsin inhibitor, 2 mM benzamidine, pH 8.2). Cells were disrupted by sonication and lysates were immediately used for phosphodiesterase (PDE) activity measurements.

PDE activities were assessed in cellular lysates as described [17] with some modifications [18].

The assay mixture (final volume 200 µl) contained 30 mM Tris HCl pH 7.4, 5 mM MgCl_2_, 0.5 µM either cAMP or cGMP as substrate including [^3^H]cAMP or [^3^H]cGMP (about 30.000 cpm per well), 100 µM EGTA, PDE isoenzyme-specific activators and inhibitors as described below and AEC-II lysate. Incubations were performed for 20 min at 37°C and reactions were terminated by adding 50 µl 0.2 M HCl per well. Assays were left on ice for 10 min and then 25 µg 5̀- nucleotidase (Crotalus atrox) was added. Following incubation for 10 min at 37°C assay mixtures were loaded on QAE-Sephadex A25 columns (1 mL bed volume). Columns were eluted with 2 mL 30 mM ammonium formiate (pH 6.0) and radioactivity in the eluate was counted. Results were corrected for blank values (measured in the presence of denatured protein) that were below 2% of total radioactivity. cAMP degradation did not exceed 25% of the amount of substrate added. The final DMSO concentration was 0.3% in all assays. Selective inhibitors and activators of PDE isoenzymes were used to determine activities of PDE families as described previously [19] with modifications. Briefly, PDE4 was calculated as the difference of PDE activities at 0.5 µM cAMP in the presence and absence of 1 µM piclamilast. The difference between piclamilast-inhibited cAMP hydrolysis in the presence and absence of 10 µM motapizone was defined as PDE3. The fraction of cGMP (0.5 µM) hydrolysis in the presence of 10 µM motapizone that was inhibited by 100 nM sildenafil reflected PDE5. At the concentrations used in the assay piclamilast (1 µM), motapizone (10 µM) and sildenafil (100 nM) completely blocked PDE4, PDE3 and PDE5 activities without interfering with activities from other PDE families. PDE1 was defined as the increment of cAMP hydrolysis (in the presence of 1 µM piclamilast and 10 µM motapizone) or cGMP hydrolysis induced by 1 mM Ca^++^ and 100 nM calmodulin. The increase of cAMP (0.5 µM) degrading activity in presence of 1 µM piclamilast and 10 µM motapizone induced by 5 µM cGMP represented PDE2.

### Quantitative Real-time Reverse Transcription Polymerase Chain Reaction (qRT-PCR)

Quantitative PCR were performed following standard protocols. Total RNA was extracted from AEC-II using RNeasy plus Mini Kit (Qiagen, Hilden, Germany) according to the manufacturer’s protocol. To obtain cDNA, RNA (max. 5 µg) was reverse transcribed using StrataScript reverse transcriptase (Stratagene, La Jolla, CA) with oligo-dT primers (Eurofins MWG Operon, Ebersberg, Germany) for 1 h at 50°C. Oligonucleotide primers specific for the individual surfactant proteins were designed according to published sequences (shown in Table 1) and were synthesized by Eurofins MWG.PCR was performed with SybrGreen Mix (Abgene, Hamburg, Germany) using an iCycler (Bio-Rad Laboratories, München, Germany) under following conditions: 95°C for 30 s, 60°C (GapDH), 59,3°C (SP-A1), 57°C (SP-A2, SP-B, SP-C and SP-D) for 30 s and 72°C for 1 min (35 cycles).

**Table 1:** Primer sequences.

Primer	Accession #	forward Sequences	reverse sequence
GapDH	NM_002046	Caccagggctgcttttaact	gatctcgctcctggaagatg
SP-A1	NM_005411	Aagcagctggaggctctgt	tgctctcactgactcacacca
SP-A2	NM_006926	gagcctgaaaagaaggagca	tccaacacaaacgtccttca
SP-B	NM_000542.3	cattttccaggacacgatga	cagggggaagtagtcgtcaa
SP-C	NM_003018.3	cctgaaacgccttcttatcg	ctccagaaccatctccgtgt
SP-D	NM_003019.4	ccaggctgctttctctcagt	ctgtgcctccgtaaatggtt

Glyceraldehyde-3-phosphate dehydrogenase (GapDH) was used for normalization. The primer efficiency for GapDH was 96%, SP-A1 112%, SP-A2 85%, SP-B 91%, SP-C 95% and SP-D 100%. A threshold cycle value (C_t_) was calculated and used to calculate the relative expression (rE) level of mRNA for each sample by using the following formula:
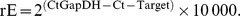



### Western Blot Analysis

Western blot analyses were performed following standard protocols.

To analyze total SP-A expression on protein levels, 3×10^6^ AEC-II were stimulated with dibutyryl cAMP or roflumilast-N-oxide at the indicated concentrations. Protein secretion was inhibited subsequently with Brefeldin A (10 µg/ml). Following the stimulation period the cells were washed once in PBS and lysed in ice cold lysis buffer. Whole cell lysates were boiled at 93°C for 5 minutes in equal volumes of loading buffer (0.5 M Tris-HCl pH 6.8, 2% SDS, 0.05% bromphenolblue, 20% 2-mercaptoethanol, 10% Glycerol). Native SP-A purified from a patient with alveolar proteinosis (kindly provided by Cordula Stamme, Borstel, Germany) was used as positive control. All samples were subjected to 12% sodium dodecylsulfate polyacrylamide gel (SDS-PAGE), separated electrophoretically and transferred to a polyvinylidene difluoride membrane (PVDF, Millipore, Schwalbach, Germany). After blocking for 2 h in Tris-buffered saline (TBS) containing 5% non-fat dry milk, the membranes were incubated with primary antibody detecting both isoforms of SP-A (rabbit polyclonal SP-A, Santa Cruz Biotechnology, Santa Cruz CA) diluted 1∶700, SP-B (goat polyclonal SP-B, Santa Cruz Biotechnology, Santa Cruz CA) 1∶200, ProSP-C (rabbit polyclonal ProSP-C, Abcam, Cambridge, UK) 1∶400 or SP-D (mouse monoclonal SP-D, Abcam, Cambridge, UK) 1∶10000 with TBS at 4°C overnight. After incubation the membrane was washed five times in TBS containing 0.1% Tween 20 (TTBS). For visualization a secondary antibody (horseradish peroxidase conjugated donkey anti-goat, Santa Cruz or IRDye 800CW conjugated donkey anti-goat, Li-COR Bioscience, Lincoln NE) was applied at a 1∶10 000-1∶20000 dilution for 2 h. After further five washes, blots were visualized using a chemiluminescent detection system (ECL, Amersham Biosciences, Freiburg, Germany) or using the Odyssey system (Li-COR Biosciences), respectively.

### Statistical Analysis

Data are presented as mean ± SD. Paired analysis was performed by Wilcoxon signed rank test, unpaired analysis by Mann-Whitney and correlations were performed using Spearman Rank Correlation Test. Probability less than 0.05 values were considered significant. Calculations were performed using STATVIEW 5.01 Version software (SAS Institute Inc., Cary, NC).

## Results

### Expression of PDE Isoenzymes in Human Primary Type II Epithelial Cells

**Figure 1:**
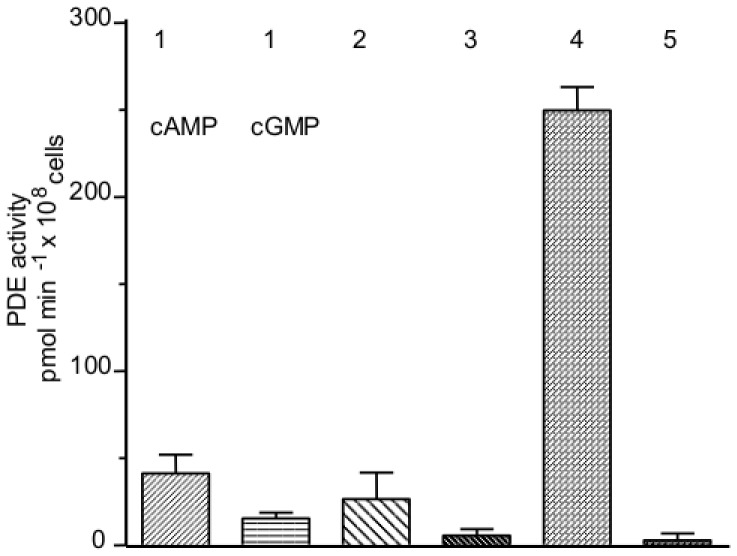
PDE1-5 activities in human AEC-II cells. Cell lysates of human AEC-II probed for PDE1-5 activities at 0.5 µM cAMP or cGMP substrate concentrations as detailed in Methods. PDE1 was measured with cAMP or cGMP as substrate. Results are shown as mean ±SEM from three different donors.

First we addressed whether PDE4 is present in primary human AEC-II: To this end lysates of AEC-II from three different donors were analyzed for cAMP- or cGMP-hydrolyzing phosphodiesterase activity in the presence or absence of selective inhibitors to dissect PDE 1–5. Under these conditions, PDE4 impresses as the major cAMP hydrolysing activity (77% of total; Figure 1) whereas cGMP-hydrolysing PDE activity was almost negligible.

### Differences in Surfactant Protein mRNA Levels in Freshly Isolated and Cultured Human AEC-II

**Figure 2:**
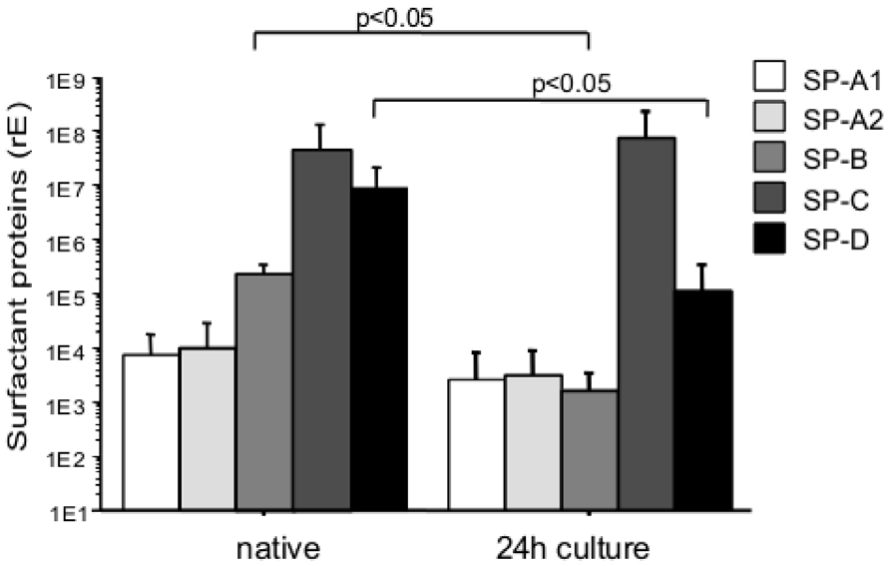
mRNA levels of surfactant proteins in human AEC-II. SP-A1, SP-A2, SP-B, SP-C and SP-D mRNA expression level of human AEC-II (freshly isolated or cultured for 24 h) were measured by real-time PCR. Bar charts show mean ± SD (n = 5, native = freshly isolated non-cultured cells; 24 h culture  =  isolated cells cultured as described in Material and Methods).

Surfactant protein mRNA levels in human AEC-II were measured before and after 24 h culture. In freshly isolated cells SP-C and SP-D mRNA was found highest compared with SP-B, SP-A2 and SP-A1. After 24 h in culture SP-B and SP-D expression was decreased significantly (p<0.05) while SP-A1, SP-A2 and SP-C mRNA expression remain constant (Figure 2).

AEC-II preparations disclosed a large variation in the mRNA expression of all types of surfactant proteins. Within the non-cultured preparations mRNA expression of SP-A1, SP-A2, SP-C, and SP-D, respectively, correlated significantly and positively with each other (p>0.95, p<0.001 in all comparisons). No correlation of SP-B mRNA expression with any of the other surfactant proteins was detected. After culture, mRNA expression of SP-C and SP-D still showed a significant and positive correlation (p = 0.9, p<0.0001), whereas the correlation of SP-A1 and SP-A2 decreased (p = 0.6, p<0.01) and no further correlation could be observed. These data suggest an unequal loss of surfactant protein mRNA expression within the culture period.

Highest expression of SP-B in the non-stimulated cultures was found in ex-smokers compared with active smokers (p<0.005, Figure S1. Interestingly, SP-B Expression increased with increasing smoking.free period (rho = 0.65, p<0.05). No significant differences between current and ex-smokers were found in the expression of the other surfactant proteins. In addition, no association of surfactant protein expression with age and sex could be found.

### Roflumilast-N-oxide Induces the Expression of SP-A1 mRNA in Human AEC-II with Low Basal Intracellular SP-A Level

**Figure 3:**
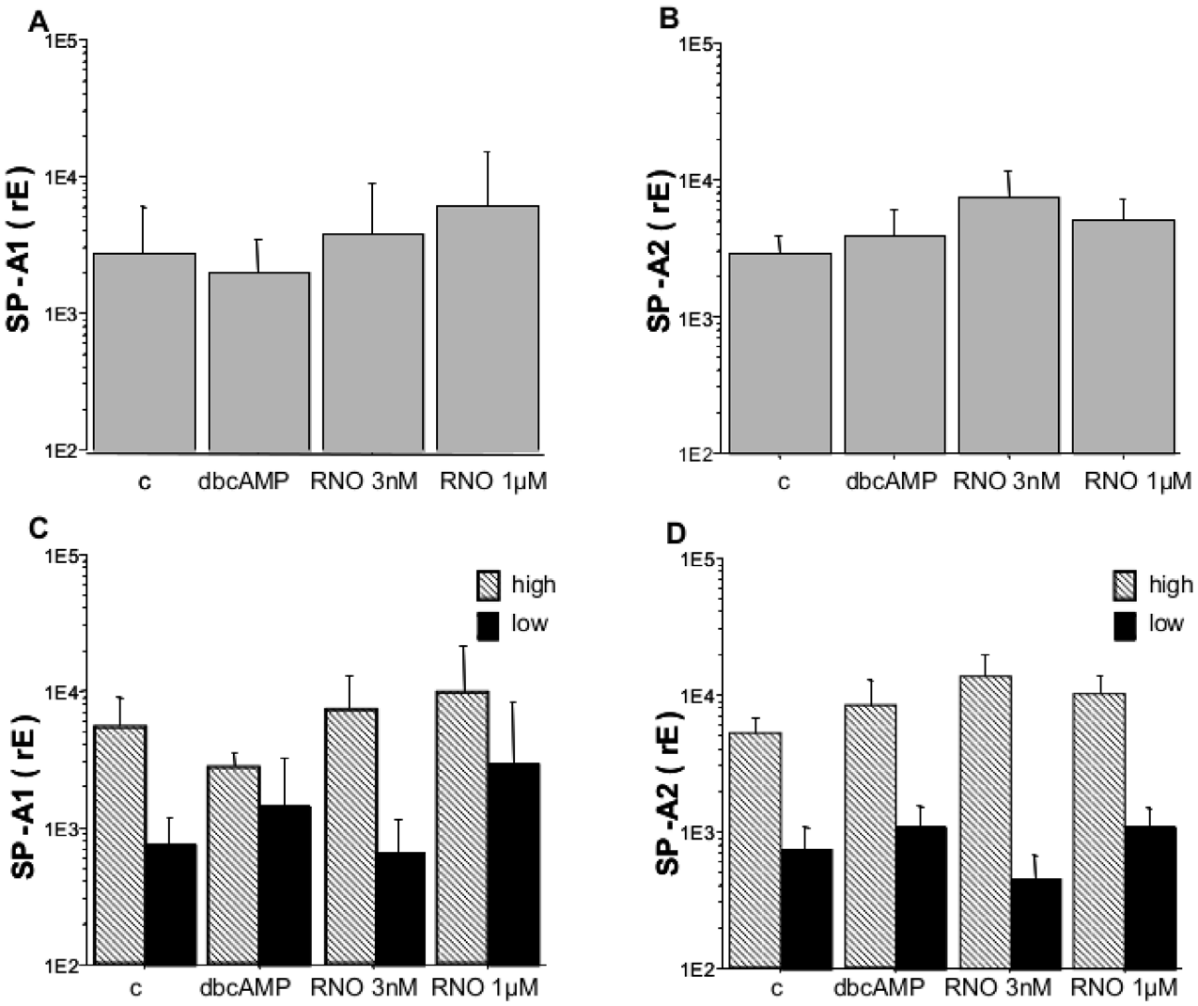
SPA-1 and SP-A2 mRNA level. Freshly isolated AEC-II were cultured for 24 h with or without the indicated substances. After the culture period expression of SP-A1 (A) and SP-A2 (B) mRNA was measured by real-time PCR. Due to the large variation in SP-A expression cultures were grouped according their basal median expression of SP-A1/SP-A2 (low level SP-A1<1436, SP-A2<2500, black bars; high level SP-A1>1436 and SP-A2>2500, hatched bars; C, D). Bar charts show mean±SD (n = 13, A; n = 13, B; n_high_ = 6, n_low_ = 7, C; n_high_ = 7, n_low_ = 6, D; rE = relative expression; c = non-stimulated control; dbcAMP = dibutyryl-cAMP; RNO = roflumilast-N-oxide).

The PDE4 inhibitor roflumilast-N-oxide was used at a concentration of 1 µM that completely and selectively inhibits PDE4 and at 3 nM which is close to the therapeutic plasma level in man [20]. Although the highest concentration of roflumilast-N-oxide doubles SP-A1 expression of AEC-II, the observed increase did not reach statistical significance. Other concentrations of roflumilast-N-oxide and dbcAMP did not exert any influence (Figure 3A and B).

Based on the median of the SP-A expression, we divided the cells in such with low (values SP-A1: rE <1436; SP-A2: rE <2500) and high baseline intracellular mRNA expression (values SP-A1: rE >1436; SP-A2: rE >2500). Regarding cells with a low baseline SP-A1 mRNA expression level, stimulation with roflumilast-N-oxide 1 µM induced an insignificant trend towards augmentation in SP-A1 mRNA expression, but not in SP-A2 mRNA (Figure 3C and D).

### Incubation of AEC-II with Indomethacin and PGE2 Allows Roflumilast-N-oxide to Increase SP-A1 and SP-A2 mRNA with a Low Intracellular Basal Level

**Figure 4:**
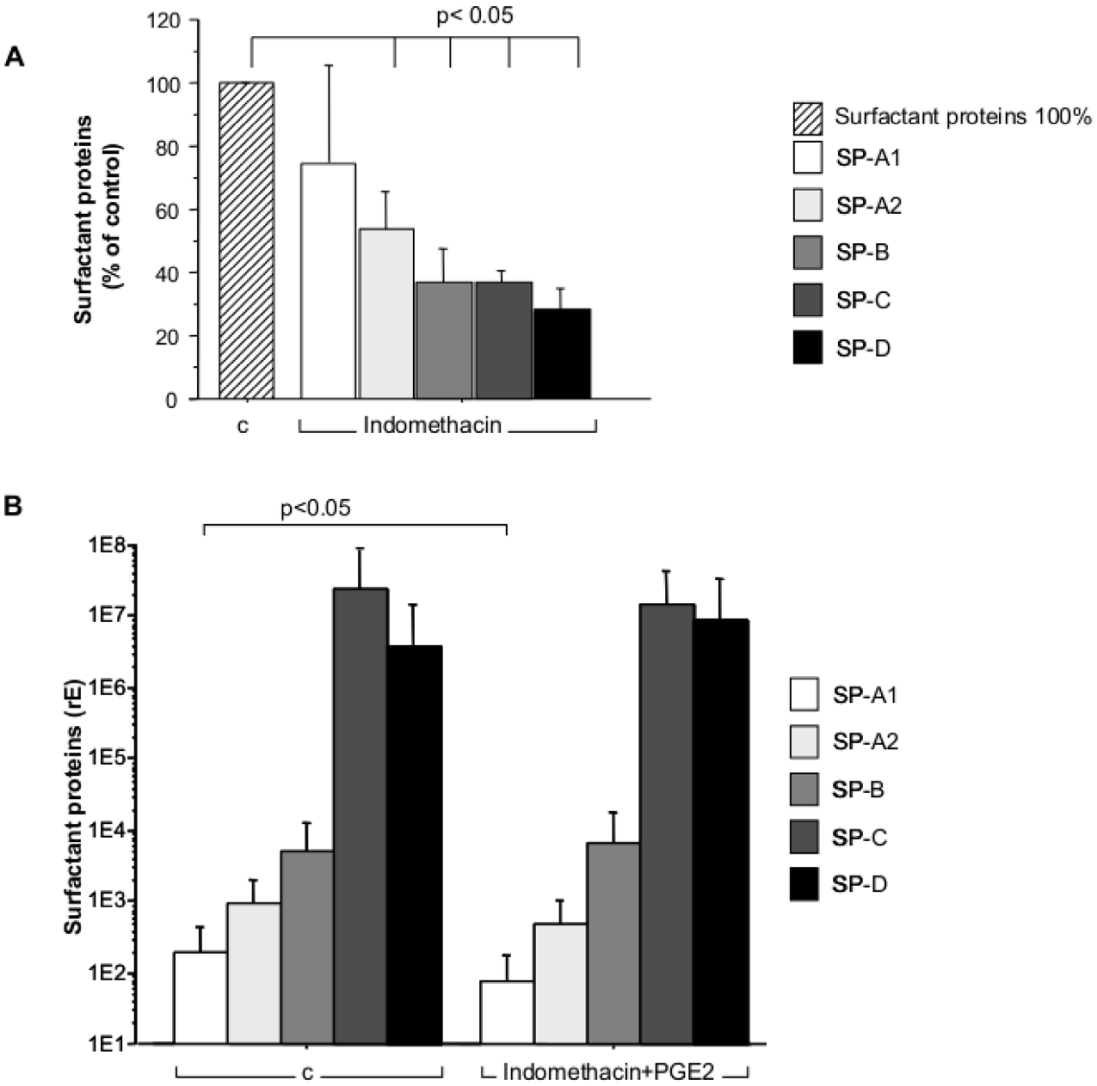
Surfactant proteins in absence or presence of indomethacin alone or in combination with PGE2. A) AECII were cultured without or with the cyclooxygenase inhibitor indomethacin (1 µM) and SP-A1, SP-A2; SP-B; SP-C and SP-D mRNA were measured by real-time PCR after culture. Relative Expression of surfactant proteins in cultures without indomethacin (C, hatched bar) were used to calculate the percentage of remaining surfactant expression after cyclooxygenase inhibition (indomethacin, n = 5)). B) Change of relative expression of surfactant proteins in cultures without (C; left panel) and with inhibition of cyclooxygenase by indomethacin (1 µM) and substitution with external PGE2 (10 nM, right panel) measured by real-time PCR. Bar charts show mean ± SD (surfactant proteins n = 8; rE = relative expression; c = control; PGE2 = prostaglandin E2).

AEC-II produce PGE2 that may act in an autocrine manner to modulate their cAMP content in a donor-dependent manner, which may in turn influence effects resulting from PDE4 inhibition. To minimize these suspected interactions related to endogenous PGE2 isolated AEC-II were preincubated with the unselective cyclooxygenase inhibitor indomethacin (1 µM). PGE2 blockade indeed reduced SP-A2, SP-B, SP-C and SP-D expression significantly by 50–70% of the controls without Indomethacin whereas the reduction in SP-A1 expression did not reach statistical significance (Figure 4A). Addition of the adenylyl cyclase activator PGE2 (10 nM) restored and standardized cAMP synthesis by the different donors. SP-B, SP-C and SP-D levels were not significantly influenced by indomethacin/PGE2, but interestingly using this protocol the expression of SP-A1 (p = 0.05) remained reduced compared with the untreated controls (Figure 4B).

**Figure 5:**
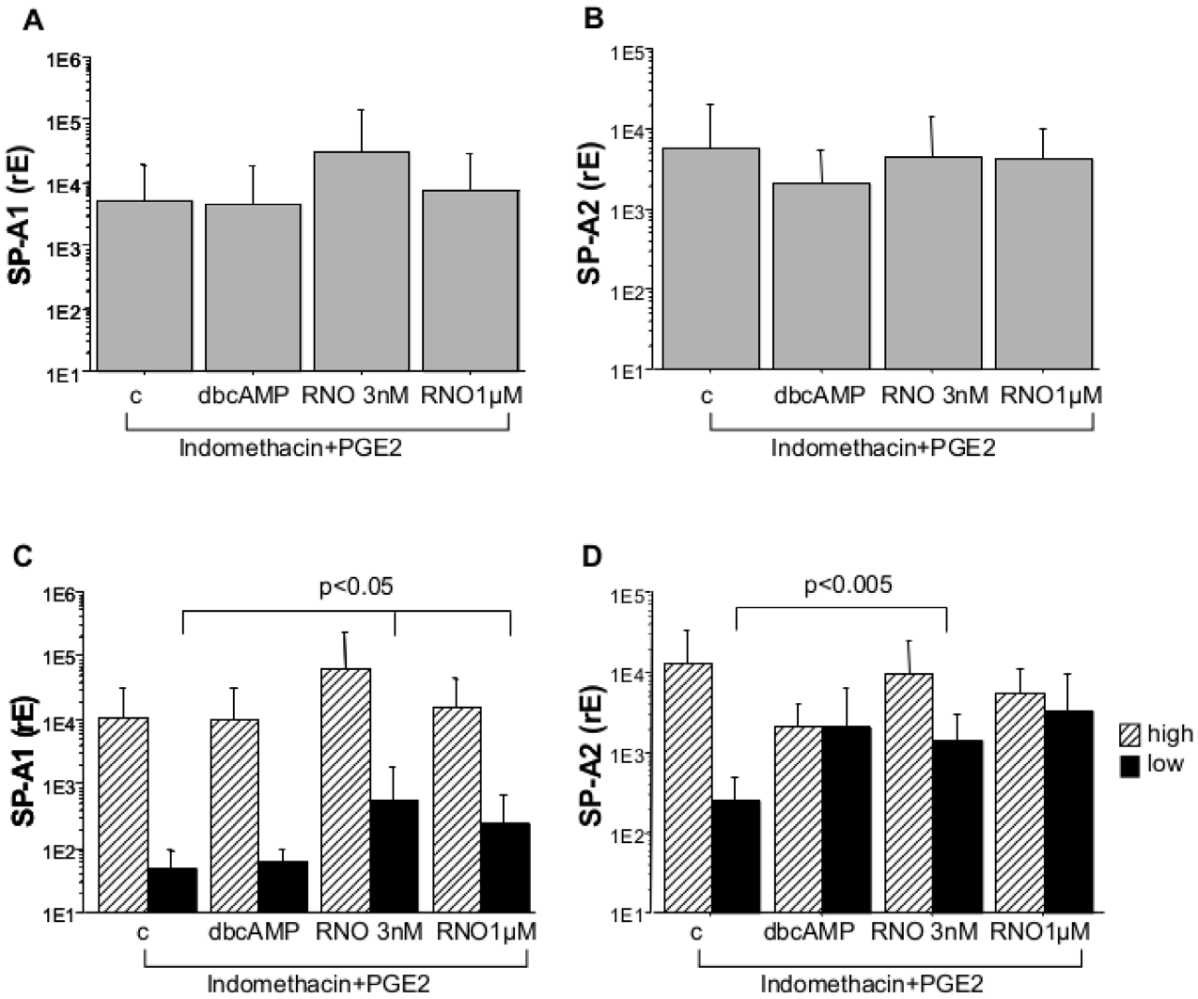
SP-A1 and SP-A2 mRNA level with indomethacin and PGE2. Cells were cultured for 24 h in presence of indomethacin (1 µM), PGE2 (10 nM) and stimulated with dbcAMP (1 mM) or roflumilast-N-oxide (3 nM or 1 µM; A, B). Cultures were split according their basal median expression of SP-A1/SP-A2 (low level SP-A1<136, SP-A2<474, black bars; high level SP-A1>136, SP-A2>474, hatched bars; C, D). Values presented are means ± SD (n = 19; A,B; n_high_ = 9, n_low_ = 10, C, D; rE = relative expression; c = non-stimulated control; dbcAMP = dibutyryl-cAMP; RNO = roflumilast-N-oxide, PGE2 = prostaglandin E2).

Regarding all AEC-II preparations, no increase in SP-A mRNA level (Figure 5A and B) could be detected following incubation with dbcAMP (1 mM) or roflumilast-N-oxide (3 nM, 1 µM) in presence of indomethacin and PGE2. Dividing the preparations by basal SP-A expression SP-A1 and SP-A2 mRNA of cells with high basal intracellular SP-A level also showed no significant increase in the respective mRNA expression when the same incubation protocol was applied. Strikingly, in AEC-II preparations with low SP-A levels incubation with roflumilast-N-oxide results in an about 11- and 5-fold (3 nM and 1 µM, respectively; p = 0.05) increase in SP-A1 mRNA expression. SP-A2 transcripts raised 5.5-fold at 3 nM concentration (p = 0.005) and 13-fold at 1 µM concentration, however, due to a higher variability the latter was not significant (Figure 5C and D). Again, these experiments were conducted in presence of indomethacin and PGE2.

### Incubation of AEC-II with Roflumilast-N-oxide After Equilibration of Intracellular cAMP Levels with Indomethacin and PGE2 Resulted in an Increase of SP-B mRNA

Applying the same protocol as described above no significant increase in SP-B mRNA expression could be observed neither in the entire group (Figure S2A) nor after dividing the preparations based on their basal median mRNA expression (Figure S2B). However, in the indomethacin/PGE2 treated cells roflumilast-N-oxide (3 nM) induced a significant increase in SP-B expression (Figure S2C; p<0.01).

**Figure 6:**
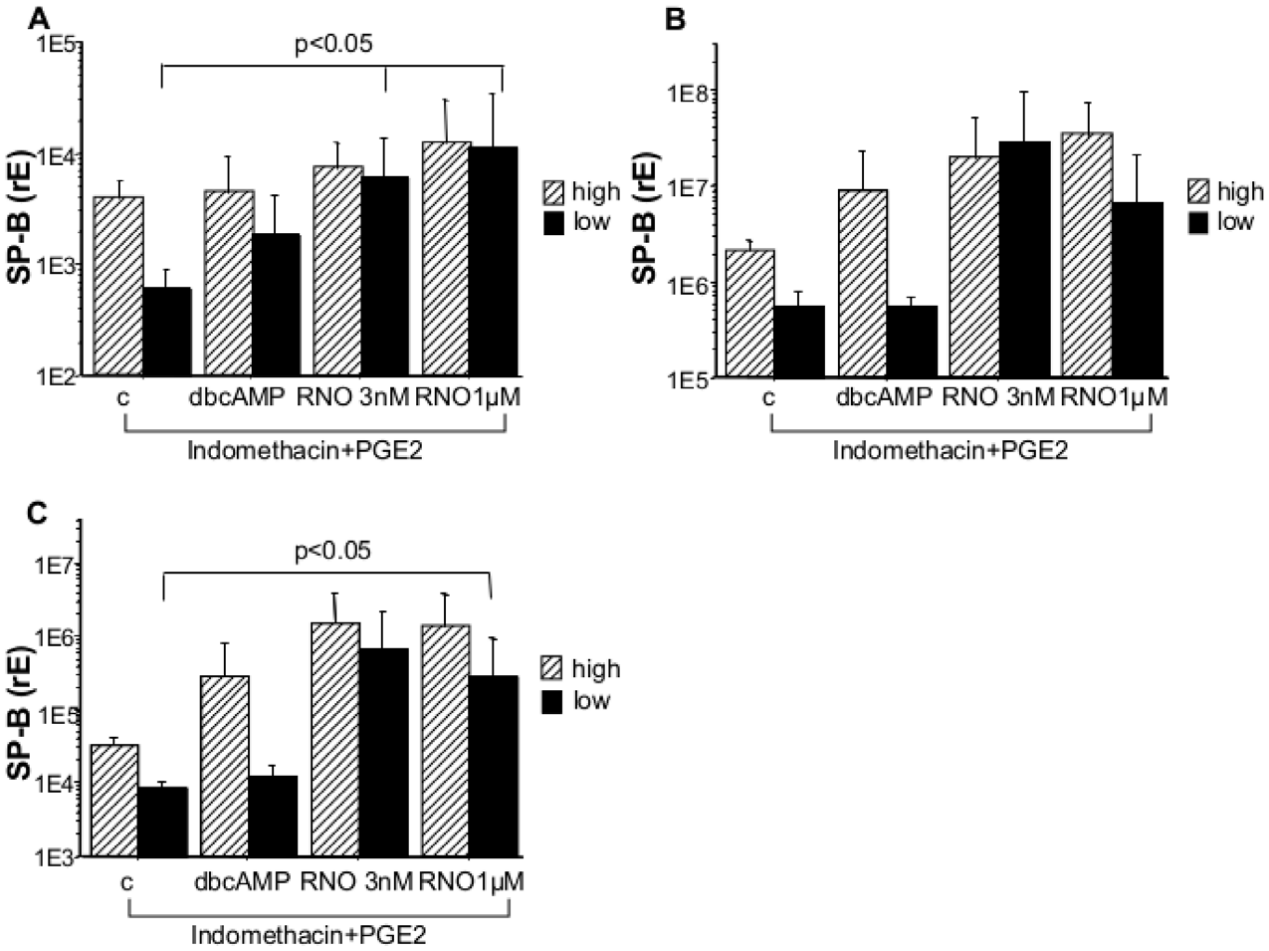
SP-B, C, and D mRNA level in presence of indomethacin and PGE2. Freshly isolated AEC-II were cultured for 24 h with indomethacin/PGE2 and the indicated substances. After the culture period SP-B (A), SP-C (B) and SP-D (C) mRNA expression was measured by real-time PCR. Due to the large variation in surfactant protein mRNA expression cultures were separated according their basal median expression of the respective surfactant protein (low level SP-B<1088, SP-C<970059, SP-D<11515 black bars; high level SP-B>1088, SP-C>970059, SP-D>11515 hatched bars; A, B, C). Bar charts show means ± SD (n_low_ = 6, n_high_ = 5, A, B,; n_low_ = 6, n_high = _6, C; c = non-stimulated control; dbcAMP = dibutyryl-cAMP; RNO = roflumilast-N-oxide; PGE2 = prostaglandin E2).

Dividing by basal median expression revealed that the overall increase in expression was mostly attributed to the low expressing preparations (Figure 6A, p<0.05). Incubation of AEC-II with roflumilast-N-oxide (3 nM and 1 µM) in presence of indomethacin and PGE2 as before increased SP-B mRNA 10-fold and 19-fold, respectively (p<0.05), in cells with a low basal intracellular SP-B mRNA level but not in high expressing cells.

### Roflumilast-N-oxide Induces SP-C mRNA Expression in AEC-II with a Low Intracellular Basal SP-C mRNA Level

Incubation of AEC-II with dbcAMP or roflumilast-N-oxide without indomethacin and PGE2 causes no changes in SP-C mRNA level (Figure S3A–C). However, equilibration of intracellular cAMP levels with indomethacin and PGE2 resulted in a 53-fold (3 nM) and 12-fold (1 µM) augmentation of SP-C mRNA in AEC-II with a low basal SP-C mRNA level (value SP-C<874791) after exposure to roflumilast-N-oxide (Figure 6B).

### AEC-II Incubated by Roflumilast Show an Increase in SP-D mRNA

Incubation of AEC-II with dbcAMP or roflumilast-N-oxide without indomethacin and PGE2 causes no changes in SP-D mRNA level (Figure S4A). However, incubation of AEC-II with dbcAMP after equilibration of intracellular cAMP levels with indomethacin and PGE2 resulted in a significant increase in SP-D mRNA (Figure S4C). Although roflumilast-N-oxide induced an even higher increase in SP-D mRNA expression, this increase did not reach statistical significance. Both, dbcAMP and roflumilast-N-oxide induced an increase in SP-D mRNA after separation of AEC-II in cells with low (SP-D<46945) basal intracellular SP-D mRNA level, however, the latter did not reach statistical significance (Figure S4B; c vs. dbcAMP p<0.05). Incubation of “low SP-D AEC-II” for 24 h with roflumilast-N-oxide in presence of indomethacin and PGE2 increased SP-D transcripts significantly by 80-fold (3 nM) and 34-fold (1 µM, p<0.05, Figure 6C).

### Total SP-A, SP-B and SP-C Protein is Enhanced After Incubation with Roflumilast-N-oxide

**Figure 7:**
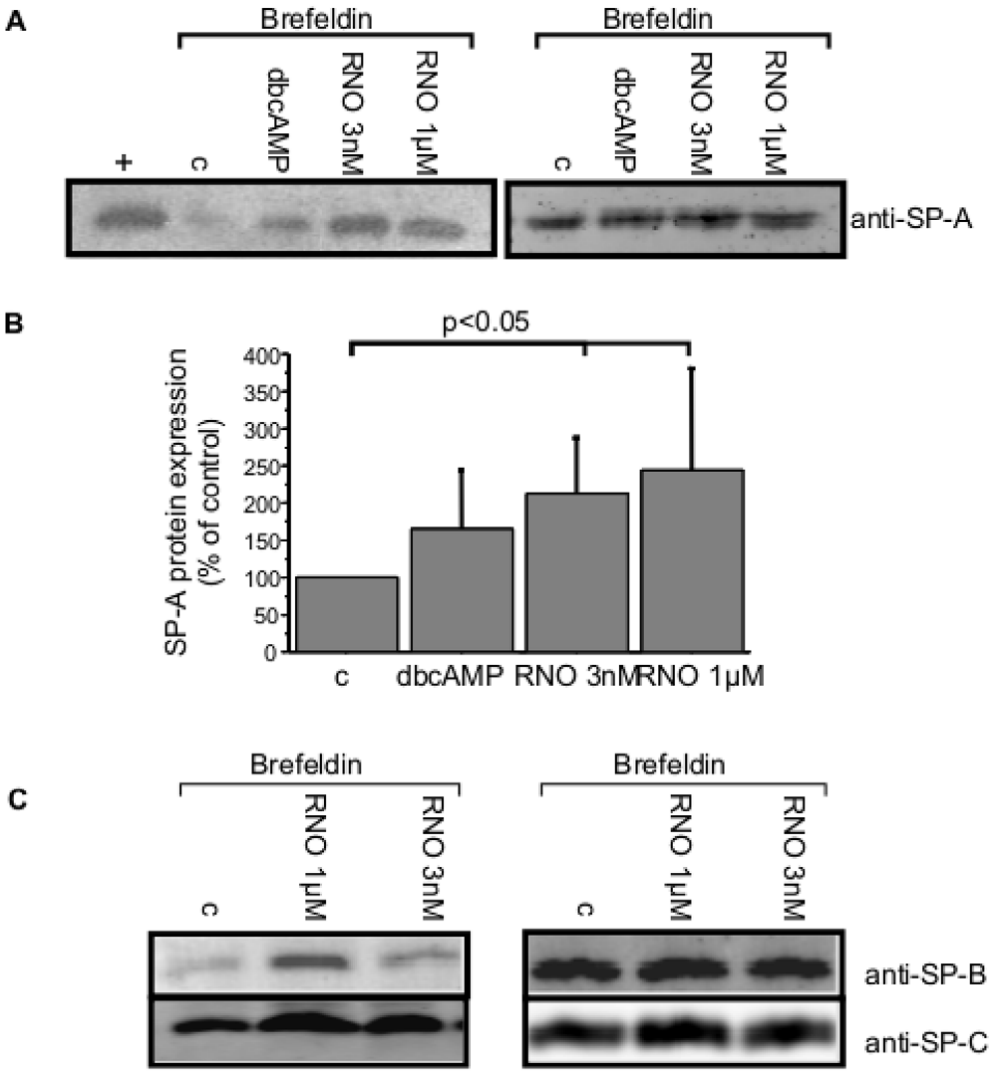
Western blot analysis of total cellular lysate of SP-A protein. Equal numbers of freshly isolated AEC-II (2×10^6^cells/well) were incubated with Brefeldin A 1 M for 15 min and stimulated with dbcAMP (1 mM), roflumilast-N-oxide at 1 µM or 3 nM. Total cell lysates were separated at reducing conditions using 12% SDS-PAGE and stained with anti-SP-A, anti-SP-B and SP-C. A: representative blot for low (left panel) and high (right panel) SP-A producer, purified SP-A from a patient with alveolar proteinosis was used as positive control, B: increase in SP-A protein expression in low SPA producers measured by densitometric analysis of the blots. Values are expressed as percentage of non-stimulated cells (n = 6), C: representative blot for low (left panel) and high (right panel) SP-B and SP-C producer. (c = non-stimulated control; dbcAMP = dibutyryl-cAMP; RNO = roflumilast-N-oxide; + = positive control, purified SP-A).

As shown above we found striking differences in SP-A mRNA expression in the different cell isolations which should be reflected by differences on protein level. Indeed, also on protein levels some cell preparations showed non-stimulated high while others disclosed low SP-A protein expression. As demonstrated by mRNA expression, SP-A production of high expressing AEC-II increased only slightly after stimulation with dbcAMP or roflumilast-N-oxide (3 nM, 1 M; Figure 7A right panel). In contrast, dbcAMP stimulation of AEC-II of low basal SP-A protein level causes an in-significant increase in SP-A protein production (p = 0.07, Figure 7A left panel). Analysis of 5 Western blots revealed a dose-dependent increase by stimulation with 3 nM or 1 µM roflumilast-N-oxide (p<0.05, respectively; Figure 7B).

The same results were observed by Western blot against SP-B and SP-C. Cell preparations with non-simulated high protein levels (Figure 7C, right panels) showed no augmentation of SP-B or SP-C. However, surfactant protein levels of cell preparations with non-stimulated low protein levels (Figure 7C, left panels) increased after stimulation with roflumilast-N-oxide 3 nM slightly and stronger with roflumilast-N-oxide 1 µM. SP-D could not be detected on Western blot.

## Discussion

Surfactant protein production is influenced by intracellular levels of cAMP mediated by increased binding of thyroid transcription factor 1 (TTF-1) and NF-κB to the TTF-1 binding element (TBE) [21]. Intracellular cAMP levels can be increased in cell culture experiments, by either activating adenylyl cyclase, by addition of cAMP analogues or by inhibiting cAMP-degrading phosphodiesterases. Because there are no data on PDE-activity in human adult AEC-II available, we analyzed PDE-activity in freshly isolated human AEC-II and found that PDE4 is the major cAMP hydrolysing PDE isoenzyme. Consequently, the question arose whether PDE4 inhibitors may modulate surfactant protein production.

We and Wang *et al.* [22] observed a highly variable mRNA expression of the various surfactant proteins in different isolations. In our experiments, isolated cells show the highest expression in SP-C and SP-D and lower expression in SP-B, SP-A1 and SP-A2. No association of surfactant protein expression with age or sex could be detected and smoking history influences only SP-B but not the other surfactant proteins. Although we used a strictly standardised procedure for cell transportation and isolation to minimize isolation-related variability, there are still numerous potential sources for variability. These variables may encompass SP-A genotype, intracellular basal cAMP levels, expression of PDE4, levels of PGE2 or inflammatory cytokines [23].

For example, human SP-A exhibits a complex regulation at genetic and transcriptional levels. SP-A 1/SP-A2 mRNA ratio varies among individuals from 0.94 to 6.8 [24], [25]. Four alleles for SP-A1 and six alleles of SP-A2 have been identified until now [26]. Some of these SP-A alleles seem to be associated with low, others with high SP-A levels. SP-A2 allele *1A0* found in patients with RDS, is associated with low SP-A mRNA levels [27]. Different SP-A levels are found in lung diseases as exemplified by higher SP-A1/SP-A2 levels in patients suffering from adenocarcinoma which correlates with tumour-grading [28], higher level in patients with alveolar proteinosis [29] or reduced levels in diseases such as the Acute Respiratory Distress Syndrome (ARDS) [30]. It has been reported that in lungs of patients afflicted with COPD the ratio of SP-A producing to total AEC-II was reduced [31]. Further possible reasons are growth factors possibly released by tumours or hypoxia which also influences surfactant expression [32]. A limitation of this study is the missing information on in-situ cytokine expression. Although the cells were isolated from macroscopically tumour-free tissue without signs of obvious inflammatory changes, the type of tumour or more probably the degree of inflammation might influence the level of surfactant proteins. Differences in cytokine expression patterns are probably able to change surfactant protein level [33], [34], TGFβ down-regulates SP-B and up-regulates α-smooth-muscle-actin (αSMA), a marker for epithelial-mesenchymal-transition [35]. Cigarette smoke increases the expression of TGFβ [36]. Thus, different expression levels of TGFβ in the lungs employed in this study might also contribute to the variation of surfactant protein expression.

A decrease of surfactant proteins in culture is a very early sign of differentiation into AECI-like cells of the cultured AEC-II [9]. It is published, that combinations of dexamethasone, keratinocyte growth factor, isobutylmethylxanthine and cAMP stabilize AECII phenotype and surfactant protein production on cells cultured for 5 days and more on collagen or matrigel [22], [37]. However, others report that AECII cultured on collagen and exposed to TGFß undergo apoptosis whereas cells cultured on matrix components undergo epithelial to mesenchymal transition due to endogenous TGFβ activation [38]. Thus, culture of AECII on coated plates would influence our experiments. Therefore, we have chosen a short-time culture period of 24 h on plastic. Even though, SP-B and SP-D mRNA expression decreased significantly within 24 h whereas SP-A1, SP-A2 and SP-C remained stable.

In our experiments, surfactant protein mRNA expression of AEC-II was differentially increased following incubation with dibutyryl cAMP or roflumilast-N-oxide. The large inter-individual variation might occlude the influence of dbcAMP or roflumilast-N-oxide on surfactant protein mRNA expression. To reduce the variation we grouped AEC-II isolates from the different donors in two groups according to the expression of higher or lower levels of surfactant protein mRNA compared to the median of the entire cohort. However, even after this separation in most cases stimulation of AEC-II with roflumilast-N-oxide or dbcAMP showed no conclusive or only an insignificant up-regulation of surfactant protein mRNA which might be attributed to inter individual variations within endogenous cAMP or PGE2-levels. AEC-II are acknowledged producers of PGE2 which may act autocrinously to augment the cAMP content of these cells [39]. Effects of PDE4 inhibitors are contingent on cAMP production; for example, a very low activity of the adenylyl cyclase may result in poor effects from PDE4 inhibition. PGE2 may up-regulate SP-transcripts by increasing cAMP. Indeed, addition of indomethacin to block endogenous PGE2 dramatically reduced the expression of most of the surfactant proteins. This is supported by an earlier observation that SP-A gene expression in human foetal lung explant cultures is compromised by indomethacin and restored by the addition of PGE2 [40]. Thus, cAMP induction by PGE2 is an important regulatory mechanism in surfactant protein expression. Certainly, the release of PGE2 from AEC-II isolates is donor specific, possibly dependent on the activity of COX enzymes finally resulting in variable cAMP contents. Hence, heterogeneous effects of PDE4 inhibition but also basal SP-A production may be seen. In an attempt to minimize this variability, endogenous PGE2 release was blocked by indomethacin and subsequently restored by exogenous addition of 10 nM PGE2 to assure a uniform activity level of the adenylyl cyclase.

Under the conditions described above, stimulation with dbcAMP or roflumilast-N-oxide caused no increase in the respective mRNA expression in AEC-II preparations with a high level of intracellular basal SP-A1 and SP-A2 mRNA expression. However, in cells with low basal intracellular surfactant protein level, in most cases SP-A mRNA expression increased by incubation with dbcAMP and significantly by exposure with roflumilast-N-oxide. Incubation of AEC-II with roflumilast-N-oxide at 3 nM, which is close to its therapeutic plasma levels, resulted in a significant increase in SP-A1 and SP-A2 mRNA in cells with a low intracellular basal level. In conclusion, in human adult AEC-II that display low basal SP-A gene expression, inhibition of PDE4 up-regulates SP-A mRNA expression.

In human foetal lung the SP-A2 gene is more sensitive to cAMP than SP-A1 [41]. In our experiments using adult human AEC-II SP-A1 mRNA was more sensitive to regulation by roflumilast-N-oxide than SP-A2 mRNA. However, in mid-gestation foetal lung the SP-A1 level is higher than SP-A2 which turns to the opposite in adult lung [42]. The lower SP-A1 level in adult lung is perhaps an explanation for the stronger up-regulation of the SP-A1 mRNA. Besides the increase in SP-A expression, incubation of AEC-II with roflumilast-N-oxide caused also a significant augmentation of SP-B mRNA in the additional presence of indomethacin and PGE2 yet as for SP-A only in cells with a low intracellular SP-B mRNA level. DbcAMP increased SP-D mRNA significantly before and after indomethacin/PGE2. SP-C is slightly and SP-D significantly up-regulated by roflumilast-N-oxide again in cells with a low basal level.

Corroborating our results on mRNA level, Western blot analysis showed also an increased protein level after incubation with dbcAMP and roflumilast-N-oxide (1 µM and 3 nM) in low basal SP-A producing AEC-II. With the exception of SP-D protein, which was not detectable on protein level, similar results were obtained after stimulation with both roflumilast-N-oxide concentrations for SP-B and SP-C protein expression. Thus, the effects of increasing cAMP levels are not only limited to the increase in mRNA but they are also translated in increased protein release.

In these experiments we used isolated human primary AEC-II of high and documented purity. The advantage of the use of purified cells is that all effects observed can be attributed to the cell type of interest. Thus, our data demonstrate that roflumilast-N-oxide directly targets AEC-II. In tissue, however, it is possible that the observed effects might be secondary to interactions with other cells; e.g. increase in cAMP in macrophages might induce mediator release which subsequently stimulates alveolar epithelial cells type II. However, this might also be a limitation of our study as in vivo such interactions of AECII with other cells might be normal. A possibilty to include cell-cell interactions might be the use of tissue culture instead of isolated cells and surfactant protein mRNA expression ex vivo might measured either by PCR or using tissue staining or in situ-hybridization [43].

Nevertheless, the results of these experiments indicate that inhibition of PDE4, the major common phosphodiesterase in AECII, modulates surfactant protein expression in culture. Regarding the manifold biophysical and immunomodulatory activities of the surfactant proteins, interventions up-regulating surfactant proteins may unfold favourable effects in a range of respiratory ailments given their role in lung physiology and innate defence also taking into account that levels of SP-A were found to be compromised in COPD [31], pneumonia [44] or ARDS [30].

In conclusion the PDE4 inhibitor roflumilast-N-oxide increased the expression of SP-A and other surfactant proteins in isolated human adult AEC-II expressing lower levels of these proteins at baseline. Considering that cAMP is well described to augment expression of the surfactant proteins in foetal AEC-II, the increase in cAMP following treatment with the PDE4 inhibitor may account for these observations. To our knowledge, this is the first report demonstrating that PDE4 inhibitors regulate surfactant protein expression in human adult AEC-II. This increase in surfactant protein expression was visible in all surfactant protein subtypes, although the increase did not reach a significant level in all cases.

## Supporting Information

Figure S1 **Surfactant protein mRNA level in AEC-II from current and ex-smokers.** Relative expression of SP-B is significantly reduced in ex-smokers (light bars, n = 6) compared with non-smokers (dark bars, n = 15). No significant differences between smokers and ex-smokers could be detected in the relative expression of SP-A1, SP-A2, SP-D, and SP-C.
(EPS)

Figure S2 **SP-B mRNA level in absence or presence of indomethacin and PGE2.** Freshly isolated AEC-II were cultured for 24 h without (A, B) or with (C) indomethacin/PGE2 and the indicated substances. After the culture period SP-B mRNA expression was measured by real-time PCR. Due to the large variation in surfactant protein mRNA expression cultures were separated according their basal median expression of SP-B (B; low level SP-B <1340, black bars; high level SP-B >1340; diagonal striped bars). Bar charts show means ± SD (n = 18, A; n_low_ = 11, n_high_ = 7, B; n_low_ = 6, n_high = _5, n = 11, C; c = non-stimulated control; dbcAMP = dibutyryl-cAMP; RNO = roflumilast-N-oxide; PGE2 = prostaglandin E2).
(EPS)

Figure S3 **SP-C mRNA level with and without indomethacin and PGE2.** Cells were incubated 24 h without (A, B) or with (C) indomethacin/PGE2 and the indicated substances. After the culture period SP-C mRNA expression was measured by real-time PCR. Due to the large variation in surfactant protein mRNA expression cultures were divided according their basal median expression of SP-C (B; low level SP-C <874791, black bars; high level SP-C >874791, diagonal striped bars). Values presented are means ± SD (n = 18, A; n_low_ = 11, n_high_ = 7, B:; n_l_ = 11, C;̧c = non-stimulated control; dbcAMP = dibutyryl-cAMP; RNO = roflumilast-N-oxide; PGE2 = prostaglandin E2).
(EPS)

Figure S4 **SP-D mRNA level with and without indomethacin and PGE2.** AEC-II were cultured for 24 h without (A, B) or with (C) indomethacin/PGE2 and the indicated substances. After 24 h of culture SP-D mRNA expression was measured by real-time PCR. Due to the large variation in surfactant protein mRNA expression cultures were grouped according their basal median expression of SP-D (B; low level SP-D <46945, black bars; high level SP-D >46945; diagonal striped bars). Bar charts show mean ± SD (n = 18, A; n_low_ = 9, n_high_ = 9, B; n = 12, C;,c = non-stimulated control; dbcAMP = dibutyryl-cAMP; RNO = roflumilast-N-oxide; PGE2 = prostaglandin E2).
(EPS)
